# First pathologically confirmed case of concurrent primary renal cell carcinoma and metastatic lung adenocarcinoma in the same kidney

**DOI:** 10.3389/fonc.2026.1767473

**Published:** 2026-02-10

**Authors:** Hua Tang, Shengchun Zhang, Jian Chen, Yongfu Wang, Changping Guo, Ting Yu, Dechuan Ye

**Affiliations:** Department of Urology, Second Hospital of Sanming City, Yongan, Fujian, China

**Keywords:** case report, lung adenocarcinoma, multiple primary cancers, renal cell carcinoma, tumor-to-tumor metastasis

## Abstract

**Background:**

The coexistence of a primary tumor and a histologically distinct metastasis within the same organ presents a rare diagnostic dilemma, challenging the classical criteria for multiple primary malignant neoplasms (MPMNs). We report the first pathologically confirmed case of synchronous primary clear cell renal cell carcinoma (RCC) and metastatic lung adenocarcinoma within the same kidney.

**Case description:**

A patient with tumors in the left lung, right femur, and left kidney underwent laparoscopic left nephrectomy. Postoperative pathology surprisingly identified two distinct lesions: a primary clear cell RCC and a metastatic adenocarcinoma. Subsequent biopsies confirmed the lung as the primary source of the adenocarcinoma (EGFR L858R mutation). The patient was treated with furmonertinib and radiotherapy.

**Conclusion:**

This case underscores the critical importance of histopathological confirmation in complex presentations. It illustrates that even with imaging suggestive of a single primary carcinoma, meticulous pathological evaluation is essential to uncover synchronous MPMNs, thereby guiding precise therapy. The patient demonstrated a positive response to targeted treatment.

## 1 Introduction

Multiple primary malignant neoplasms (MPMNs) are two or more histologically distinct primary cancers present in one person (1). MPMNs in kidneys are particularly challenging because to identify multiple renal tumors three different types of tumors must be distinguished: (1) synchronous primary renal tumors, (2) one renal primary with intraparenchymal metastasis, (3) rare tumor-to-tumor metastasis (TTM) where the metastasis colonizes an existing renal tumor (2). The Warren and Gates criteria establish the pathological basis for this differentiation (1). Although coexisting primary lung cancer and renal cell carcinoma (RCC) have been reported (3, 4) and renal metastases from lung adenocarcinoma have been recognized (5), the case of pathologically confirmed primary clear cell RCC coexisting with metastatic lung adenocarcinoma in the ipsilateral kidney has not been reported. This is the first case, emphasizing the diagnostic and therapeutic challenges associated with this case, and the importance of careful pathology in care.

## 2 Case description

### 2.1 Presenting complaints

**Figure 1:**
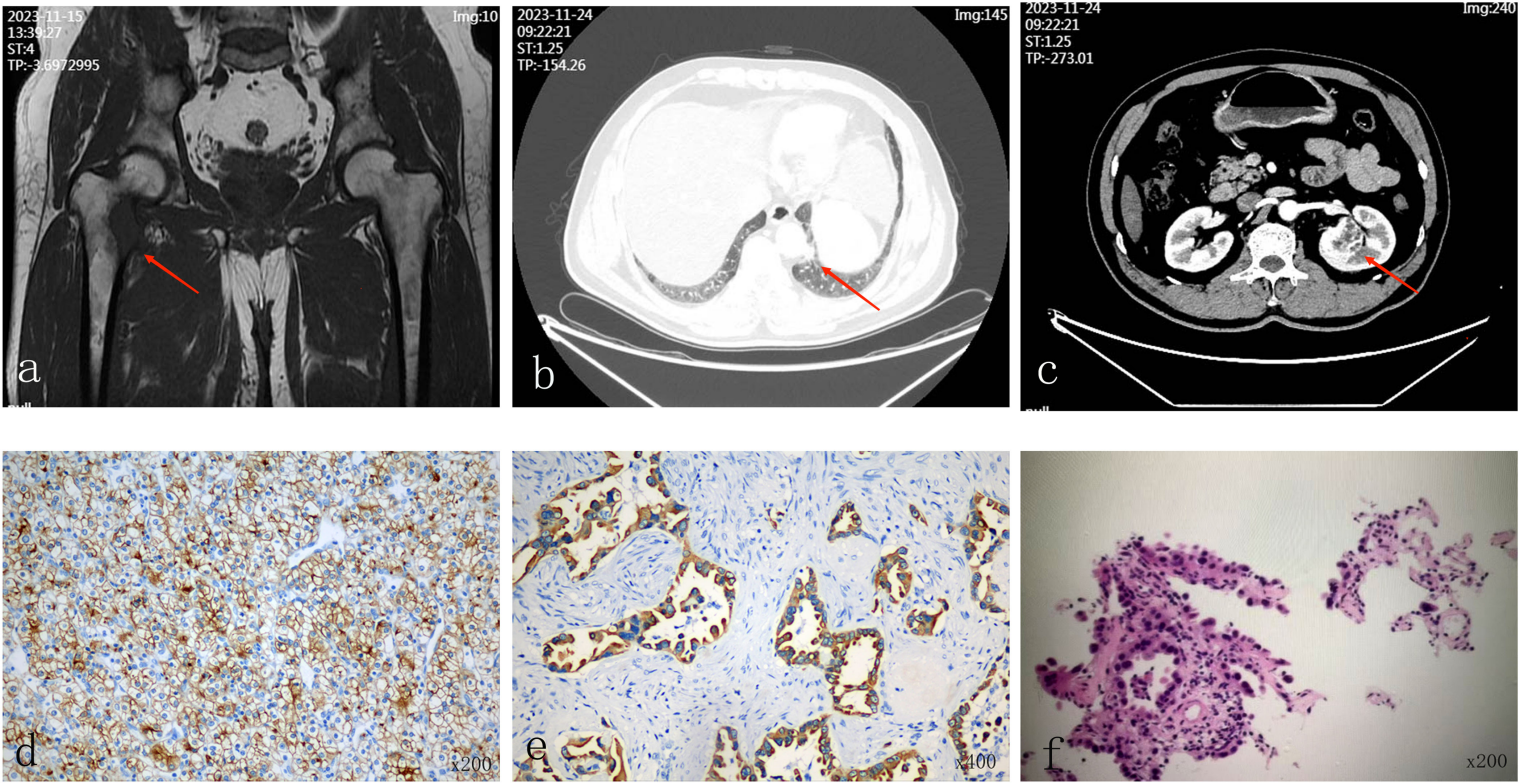
**(a)** Magnetic resonance imaging of the hip shows an abnormal signal lesion on the medial side of the proximal right femur, with surrounding muscular and soft tissue swelling and exudation (arrow). **(b)** Computed tomography (CT) of the chest demonstrates multiple solid pulmonary nodules in the left lung, with the largest lesion in the lower lobe measuring approximately 1.3 × 1.0 cm (arrow). **(c)** Contrast-enhanced abdominal CT scan reveals a slightly hypodense, heterogeneous mass in the mid-pole parenchyma of the left kidney, approximately 3.9 × 3.5 cm in size (arrow). **(d)** Hematoxylin and eosin (H&E) staining of the left renal tumor showing clear cell RCC with strong CD10 expression (IHC, ×200). **(e)** H&E staining of the same renal specimen reveals adenocarcinoma morphology with high CK7 expression (IHC, ×400). **(f)** Biopsy of the left lung lesion confirms pulmonary adenocarcinoma (IHC, ×200).

Informed consent was obtained, and all personal identifiers were removed to ensure patient confidentiality. The patient was a 58-year-old male with a body mass index of 25.4 kg/m². He had no history of hypertension or diabetes. He denied any history of tobacco smoking, alcohol abuse, or significant occupational exposure to carcinogens such as asbestos or heavy metals. There was no known family history of malignancy. He reported no constitutional symptoms such as unintentional weight loss, fever, or night sweats prior to the onset of hip pain. He was admitted to the Orthopedics Department on November 14, 2023, with the chief complaint of right hip pain for 10 days. Magnetic resonance imaging of the right hip was performed to evaluate the pain (**Figure 1a**). For systemic staging, contrast-enhanced computed tomography (CT) of the chest and abdomen was conducted. The CT scan revealed multiple solid nodules in the left lung (**Figure 1b**) and a solitary solid mass in the mid-pole of the left kidney (**Figure 1c**).

### 2.2 Radiologic and clinical staging

The contrast-enhanced CT findings indicated that the renal mass measured approximately 3.5 cm and was situated at the mid-pole of the left kidney. This mass exhibited characteristics suggestive of primary renal malignancy, including a well-defined, solid enhancing pattern. In contrast, the pulmonary lesion presented as multiple solid nodules lacking invasive features, while the femoral lesion appeared lytic without evidence of true extraosseous tumor extension; the surrounding soft tissue changes were interpreted as reactive edema, findings that were considered more consistent with metastatic disease rather than a primary bone tumor. Consequently, the working diagnosis at presentation favored a primary renal tumor with metastases to the lung and bone. The clinical stage at that time was estimated as cT1aN0M1.

### 2.3 Therapeutic intervention

Although the lung lesion was not confirmed before surgery, radical nephrectomy was performed in the interest of diagnosis and cytoreduction. Both preoperative renal biopsy and intraoperative frozen section were skipped, due to the disagreement among the Multidisciplinary Team (MDT). The MDT agreed that renal mass showed typical imaging characteristics of clear cell RCC, such as well-circumscribed margins and heterogeneous enhancement. Since renal mass was centrally located and clinically relevant to oligometastatic disease, radical nephrectomy was considered the most effective and definitive approach for diagnostic certainty and potential therapeutic benefit, while minimizing the risks and sampling limitations of biopsy. After informed consent from the patient and his legal representative, retroperitoneal laparoscopic radical left nephrectomy was performed under general anesthesia on November 29. Nephron-sparing surgery was considered inappropriate due to tumor location and priority for cytoreduction. Postoperative pathology revealed two distinct tumors in the left kidney. Grossly, a yellow-tan, well-circumscribed solid mass measuring approximately 3.5 cm was located at the mid-pole of the kidney. Adjacent to this lesion, a separate, firm, grayish-white nodule measuring about 0.7 cm was identified. Microscopically, the larger lesion was composed of clear cells arranged in nests and acini, consistent with clear cell RCC classified according to the 2022 World Health Organization Classification of Urinary and Male Genital Tumors, International Society of Urological Pathology grade 2. Immunohistochemical staining was positive for PAX-8 and CD10, supporting a renal origin (**Figure 1d**). The smaller lesion exhibited glandular structures with mucin production and nuclear atypia. Immunohistochemistry showed strong positivity for CK7, TTF-1 and Napsin A, confirming the diagnosis of metastatic pulmonary adenocarcinoma (**Figure 1e**). To further characterize the primary and metastatic components, the patient underwent CT-guided biopsy of both the upper segment of the right femoral tumor and the left lung tumor. The biopsy results confirmed lung origin in both cases. Genetic testing revealed an Epidermal Growth Factor Receptor (EGFR) exon 21 L858R mutation in the lung tumor tissue detected via PCR-based analysis (**Figure 1f**). No next-generation sequencing was performed, as the PCR-based identification of the EGFR exon 21 L858R mutation provided a clear, actionable target for first-line targeted therapy.

### 2.4 Final diagnosis

1. Left lower lobe adenocarcinoma with metastases to the left kidney and right femur, cT1bN0M1c, Stage IVB (EGFR L858R mutation).

2. Left kidney clear cell carcinoma, pT1aN0M0, Stage I.

### 2.5 Outcome and follow-up

**Figure 2:**
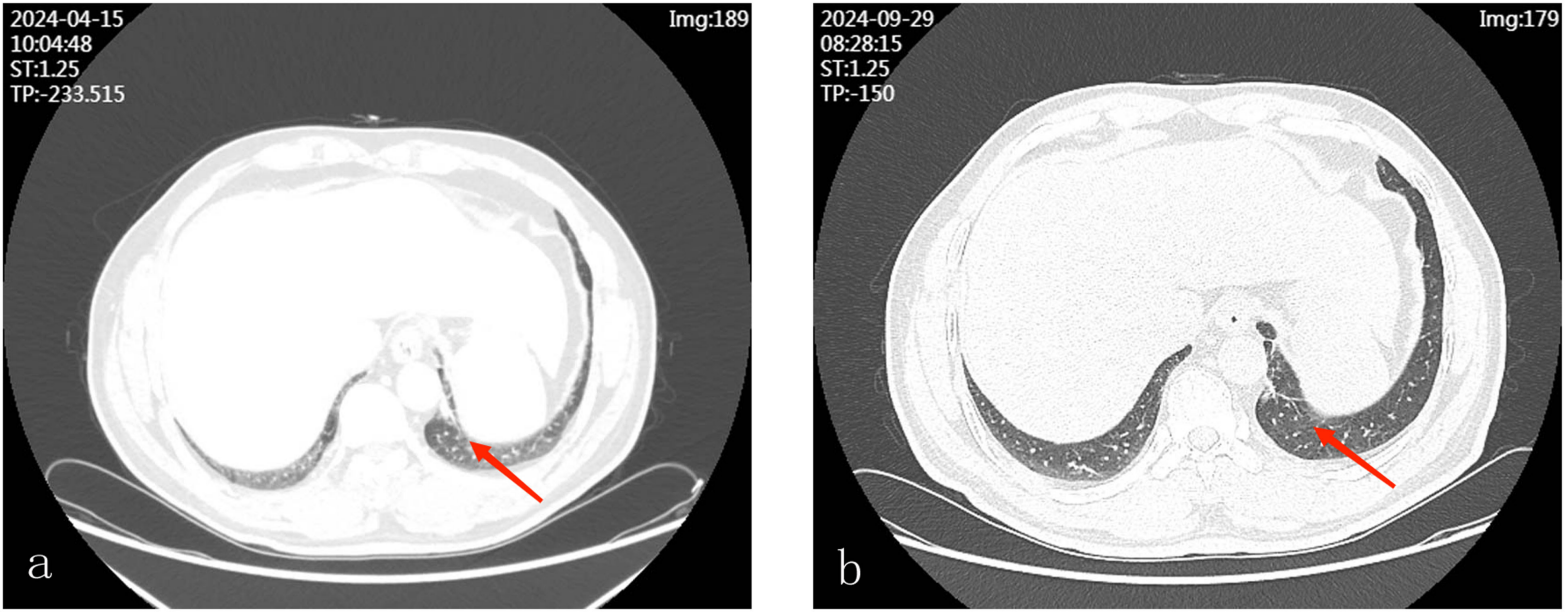
**(a)** Follow-up chest CT two months after targeted therapy shows a reduction in the size of the solid nodule in the lower lobe of the left lung, now approximately 0.7 × 0.7 cm. **(b)** Follow-up chest CT seven months after treatment shows near-complete resolution of the left lung nodule.

**Figure 3:**
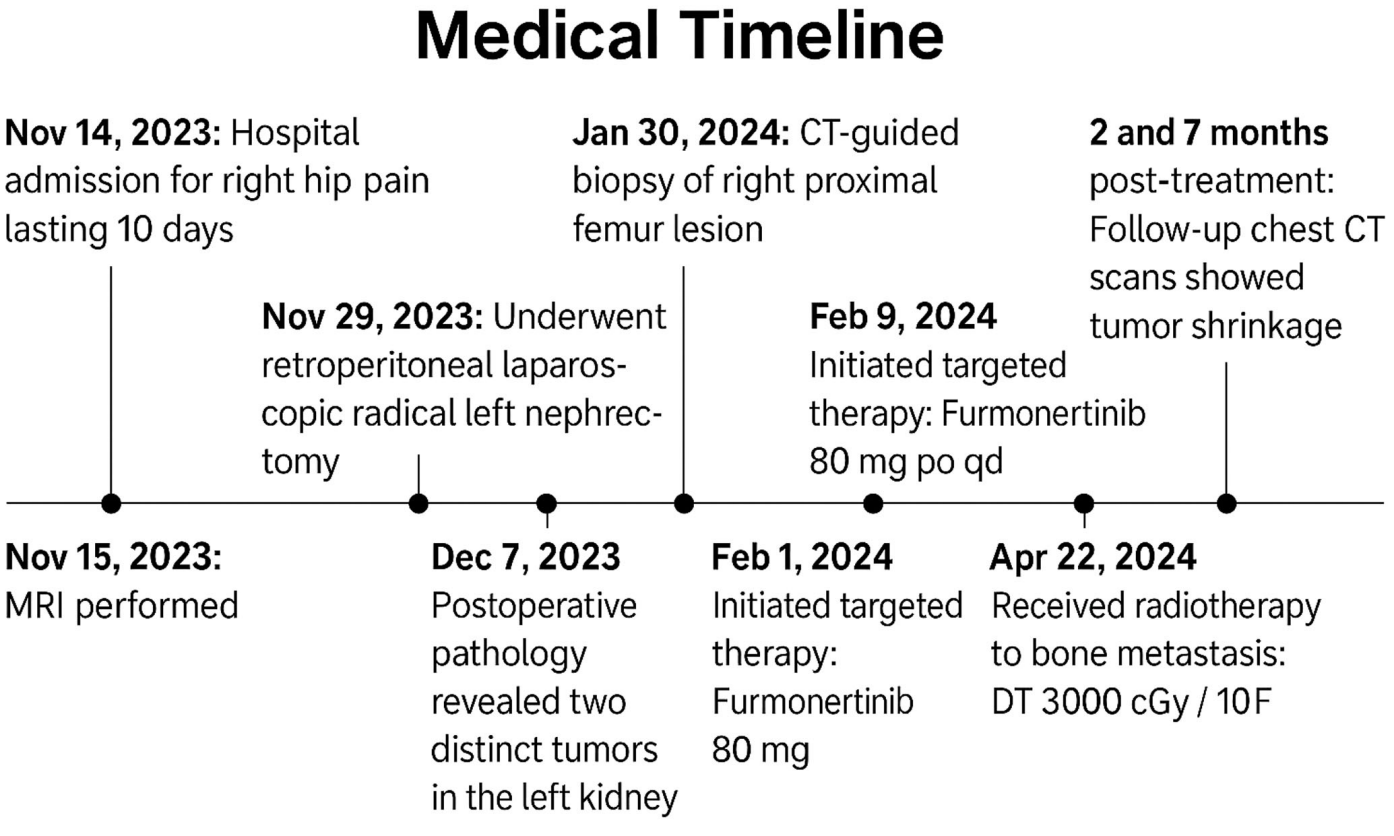
Medical timeline of the patient’s clinical course. The timeline summarizes key events from initial hospital admission for right hip pain on November 14, 2023, through diagnostic imaging, surgery, pathological confirmation of two distinct tumors in the left kidney, initiation of targeted therapy with Furmonertinib, radiotherapy for bone metastasis, and follow-up chest CT scans at 2 and 7 months post-treatment showing tumor shrinkage.

The patient began targeted therapy with Furmonertinib 80 mg orally once daily on February 9, 2024, and received radiotherapy for bone metastasis on April 22, 2024 (DT 3000 cGy/10F). Follow-up chest CT scans at 2 and 7 months post-treatment demonstrated tumor reduction (**Figure 2**). To help visualize the complex sequence of events, we created a timeline of major clinical milestones (**Figure 3**).

## 3 Discussion

This case illustrates the common but clinically important diagnostic problem that arises when multiple tumors are identified in different organs. When first analyzed, the renal mass had a radiographicly dominant presence, with a well-defined enhancement pattern typical of clear cell RCC, and clinicians favored a working diagnosis of primary renal malignancy with systemic metastases. In contrast, the pulmonary lesions were small, non-invasive and had no obvious malignant features on imaging, and were less likely to be considered primary tumor on the first examining. This difference in radiologic prominence illustrates how the overreliance on imaging characteristics can skew the diagnostic decision and delay the consideration of synchronous malignancy, especially in patients with multifocal disease identified at staging examinations (6).

The definitive diagnosis of this case was based on the Warren and Gates criteria for MPMNs (1): first, histopathological examination showed that both renal lesions were clearly malignant; second, tumors were different in structure: one consisted of clear cells nestled in nests and acini; the other constituted gland-forming adenocarcinoma producing mucin; third, immunohistochemistry found that the clear cells tumor had renal markers (PAX8, CD10), and the adenocarcinoma had pulmonary markers (TTF-1, Napsin A).

The coexistence of two tumors within the same kidney necessitates careful consideration of TTM, a rare but recognized phenomenon. RCC frequently serves as the recipient tumor due to its rich vascular supply and high metabolic activity (7). However, reported cases of TTM typically exhibit histological intermingling or infiltration of metastatic tumor cells within the host neoplasm (8). In the present case, the two tumors were spatially distinct, exhibited no histological admixture, and were separated by normal renal parenchyma. This clear anatomical and histological separation strongly argues against TTM and supports the interpretation that the renal lesions represent independent pathological processes. Next-generation sequencing of the two renal lesions was not performed because of limited tissue availability and technical constraints, which is acknowledged as a limitation of this case.

The decision to proceed to radical nephrectomy without a biopsy is a careful decision. In general, percutaneous renal biopsy is recommended to confirm histology before starting systemic therapy for suspected metastatic disease, but in this case the renal mass was deeply endophytic and centrally located, causing hemorrhage, inadequate sampling false-negative results (9). After a thorough evaluation by a MDT, surgery was chosen to provide diagnostic certainty and cytoreductive benefits. Note that the evidence supporting cytoreductive nephrectomy is heterogeneous and only for a select group of patients (10, 11). Consequently, this management approach should be viewed as tailored to individual circumstances rather than universally applicable.

Molecular characterization of lung adenocarcinoma is crucial for guiding systemic therapy. The identification of an EGFR exon 21 L858R mutation serves as a definitive indication for first-line treatment with EGFR tyrosine kinase inhibitors (12). Prior studies have demonstrated that EGFR-mutant non-small cell lung cancers can exhibit early hematogenous dissemination, even when the primary tumor is small. This observation provides a biologically plausible explanation for the presence of renal and bone metastases in this patient (13). Consequently, this finding highlights the significance of molecular profiling in situations where the tumor burden and metastatic behavior are disproportionate to the size of the primary tumor.

While the patient exhibited a favorable radiographic response to targeted therapy during follow-up, the observation period was limited, preventing any conclusions about long-term oncologic outcomes from a single case. Synchronous MPMNs are characterized by complex clinical trajectories and varied prognoses (14). Therefore, the therapeutic response noted in this instance should be interpreted with caution, serving primarily to support diagnostic accuracy rather than to provide evidence of treatment efficacy in comparable cases.

## 4 Conclusion

This case illustrates that dependence on imaging alone can obscure the presence of synchronous malignancies. When imaging results are discordant or atypical, it is crucial to obtain histopathological and molecular confirmation from multiple sites to prevent diagnostic oversimplification. Acknowledging these complex presentations can significantly impact treatment strategies and highlights the necessity of multidisciplinary decision-making in oncologic care.

## Data Availability

The original contributions presented in the study are included in the article/supplementary material. Further inquiries can be directed to the corresponding author.
